# Prevalence of Hysterectomy by Self-Reported Disability Among Canadian Women: Findings from a National Cross-Sectional Survey

**DOI:** 10.1089/whr.2021.0069

**Published:** 2021-11-29

**Authors:** Natalie V. Scime, Hilary K. Brown, Amy Metcalfe, Erin A. Brennand

**Affiliations:** ^1^ Department of Community Health Sciences, University of Calgary, Calgary, Alberta, Canada.; ^2^ Department of Health and Society, University of Toronto Scarborough, Toronto, Ontario, Canada.; ^3^ Department of Obstetrics and Gynaecology, University of Calgary, Calgary, Alberta, Canada.; ^4^ Department of Medicine, University of Calgary, Calgary, Alberta, Canada.

**Keywords:** hysterectomy, disability, functional limitations, health surveys, women's health, epidemiology

## Abstract

***Introduction:*** Our objective was to investigate differences in prevalence of hysterectomy by self-reported disability status among Canadian women.

***Materials and Methods:*** We analyzed cross-sectional data from the Canadian Community Health Survey 2012 on 30,170 women aged ≥20 years. Disability was defined as reports of sometimes or often (vs. never) experiencing functional limitations or reduction in daily activities at home, school, or work. Frequency of these limitations was used as a proxy for disability severity. The outcome was self-reported hysterectomy status. Modified Poisson regression was used to quantify the prevalence ratio (PR) and 95% confidence interval (CI) for hysterectomy according to any, functional, or activity-limiting disability, after adjustment for household income, employment, education, ethnicity, and marital status. Results were stratified by age at time of data collection, categorized as childbearing (20–44 years), perimenopausal (45–59 years), and postmenopausal (60 years and older).

***Results:*** Disability was significantly and consistently associated with higher prevalence of hysterectomy in women. The strength of association was inversely related to age category, and PRs for a given age category were similar across disability types and severity levels. PRs for the association between any disability and hysterectomy were 2.18 (95% CI 1.36–3.50) for childbearing-aged women, 1.48 (95% CI 1.21–1.80) for perimenopausal women, and 1.12 (95% CI 1.02–1.24) for postmenopausal women.

***Conclusions:*** Prevalence of hysterectomy is disproportionately higher among women with self-reported disabilities compared with women without disabilities, with these differences most pronounced in women of childbearing age.

## Introduction

Hysterectomy is one of the most commonly performed surgeries in females.^1^ Approximately 90% are performed for noncancerous gynecologic conditions,^2^ including leiomyomas, endometriosis, and pelvic organ prolapse, which can significantly reduce quality of life.^3,4^ Annual hysterectomy rates have declined over recent decades.^2^ In Canada, age-standardized rates fell from 446/100,000 in 2000 to 288/100,000 in 2019.^5^ Development of less invasive treatments and patient preferences for nonsurgical management appear to be driving this declining trend.^6^ Judicious use of hysterectomy is important, as long-term considerations for this surgery include higher associated risk of cardiovascular events and mortality, particularly when performed during reproductive years.^7^

Although the overall volume of hysterectomy is decreasing, there are persistent sociodemographic disparities in who undergoes this surgery. Hysterectomy rates are disproportionately higher among women with low education, rural residency, and who are Black.^8,9^ Delayed care-seeking until later in the disease process, lack of drug coverage for pharmacological management options, and unconscious physician bias may all be contributing factors.^8,9^ These findings have generated awareness of important inequities in gynecologic health, and prompted larger inquiries into the magnitude of these disparities.

The role of disability in hysterectomy rates has received little attention. Disability is umbrella term for functional impairments and activity limitations related to an individual's health condition and their environment,^10^ and is emerging as an important determinant of women's health. Women with disability less often use long-acting reversible contraceptive methods,^11^ more frequently experience unintended pregnancy,^12^ have heightened risk of maternal morbidity,^13^ and less often have up-to-date breast and cervical cancer screening.^14^ These differences are not fully explained by comorbidities or socioeconomic factors.

In one U.S. study, women with multiple disabilities were more likely to have had a hysterectomy (adjusted hazard ratio 1.30, 95% confidence interval [CI] 1.20–1.42), and this association was predominantly observed among women aged 20–45 years.^15^ Given differences in health care delivery between the U.S. and Canada, such as health insurance coverage, physicians per capita, and geographic density, it is unclear whether a similar disparity in hysterectomy rates by disability status exists in Canada. Therefore, our objective was to investigate differences in prevalence of hysterectomy by self-reported disability status using data from Canada's national health surveillance survey program.

## Materials and Methods

We used cross-sectional data from the 2012 Canadian Community Health Survey (CCHS) public use microdata file.^16^ The CCHS is an annual pan-Canadian surveillance program carried out by Statistics Canada (a federal government agency) that gathers information on health care use, health status, and social and personal determinants of health. The sampling frame includes individuals in the Canadian household population, and excludes individuals living on reserves or in institutions, and full-time members of the Canadian Armed Forces (<3% of the population).

Respondents are randomly sampled using multistaged stratified procedures across all provinces and territories. Data are collected through self-reported surveys administered using computer-assisted interviews. Surveys are reviewed and modified with each cycle, and consist of annual content collected on all respondents and optional content collected on respondents from a subset of provinces and territories. Questions regarding hysterectomy were most recently included in the annual content for the 2012 CCHS cycle; thereafter questions on hysterectomy were optional content or omitted.

Thus, to ensure our results were nationally representative, we used only the 2012 cycle for this analysis. Further details on the CCHS sampling and data collection methodology are available from Statistics Canada.^17^ In total, 71,614 out of 92,682 eligible households (77.3% response rate) and 62,103 individuals from each of those responding households (86.7% response rate) participated in the 2012 CCHS.^17^ The sample for this analysis were women aged 20 years and older who had complete data for hysterectomy and disability status. In accordance with the Canadian Tri-Council Policy Statement Article 2.2, this secondary analysis using publicly available CCHS data was exempted from ethical review and approval.^18^

### Variables

Disability status, and specifically long-term disability, was captured using two measures that correspond to the “Participation and Activities” domain of disability using the World Health Organization (WHO) International Classification of Functioning, Disability and Health (ICF) framework.^10^

Participants were first given the preamble: “The next few questions deal with any current limitations in your daily activities caused by a long-term health condition or problem. In these questions a ‘long-term condition’ refers to a condition that is expected to last or has already lasted 6 months of more.” Functional disability was measured by the question “Do you have any difficulty hearing, seeing, communicating, walking, climbing stairs, bending, learning or doing any similar activities?” with response options “sometimes,” “often,” or “never.”

We classified responses of “sometimes” or “often” as indicative of a functional disability. Activity-limiting disability was measured by the question “Does a long-term physical condition or mental condition or health problem, reduce the amount or the kind of activity you can do [in this setting]?” for four settings (home, work, school, or other), with response options “sometimes,” “often,” or “never.” We classified responses of “sometimes” or “often” for any of the four settings indicative of activity-limiting disability. Any disability was defined as the presence of functional and/or an activity-limiting disability.

We also developed variables for the severity of functional, activity-limiting, and any disability using frequency of limitations as a proxy, as has been done previously,^19^ where responses of “sometimes” were categorized as moderate disability and “often” were categorized as severe disability. Severity in this context thus refers to the extent to which disability interferes with one's daily activities, and not clinical severity. Data on specific diagnoses responsible for the disability and duration of disability were not available; however, participants reporting any degree of disability were asked which of the following best described the underlying condition: injury, disease/illness, aging, existed at birth, or other.

Hysterectomy was measured by the question “Have you had a hysterectomy (in other words, has your uterus been removed)?” Data on the timing or indication for hysterectomy were not available.

Covariates thought to impact the association between disability and hysterectomy were selected through a combination of literature review and subject matter expertise, and included household income quintile derived at the provincial level, highest level of education (less than high school, high school or some postsecondary, or postsecondary certificate), employment status (full-time at a business, part-time at a business, self-employed, or not employed), ethnicity (White, visible minority; derived by Statistics Canada using self-reported race and ethnicity), marital status (single, married/common-law, or divorced/widowed), body mass index (underweight, normal weight, overweight, or obese), and professionally diagnosed depression or anxiety.

To address the temporal limitation of this cross-sectional data source, we divided women into age groups demarcated by reproductive health stages of childbearing (20–44 years), perimenopausal (45–59 years), and postmenopausal (≥60 years). Because hysterectomy rates are highest among women aged 45–54 years,^20^ our reasoning was that disability was more likely to predate hysterectomy for women in the youngest group, whereas for women in the older groups there was a higher chance that disability was acquired after hysterectomy due to comorbidities and impairments that accumulate with the aging process.^21^

### Statistical analyses

We compared characteristics of women with any disability and no disability using proportions and standardized differences. Poisson regression with robust variance was used to quantify the prevalence ratio (PR) and 95% CI for hysterectomy according to any, functional, and activity-limiting disability. Models were adjusted for income, education, minority status, marital status, and employment, and included an age*disability interaction term to enable estimation of age-stratified PRs. Model equations and coefficient combinations are detailed in Supplementary Table S1.

We conducted a sensitivity analysis to examine the impact of missing data using multiple imputation with chained equations. Missing covariates were imputed for 10 data sets using STATA's mi package, and auxiliary variables available in the CCHS were included in imputation model if they demonstrated an absolute correlation of >0.1 with the covariate being imputed.^22^ We repeated our main analysis among imputed data sets to obtain pooled effect estimates. For all analyses, we applied sampling weights derived by Statistics Canada, which account for the complex survey design (*i.e.*, probability of selection and nonresponse).

Use of sampling weights enables accurate weighted point estimates, but conservative variance estimates. Data cleaning and analysis were carried out in STATA IC Version 15.

## Results

**FIG. 1.**
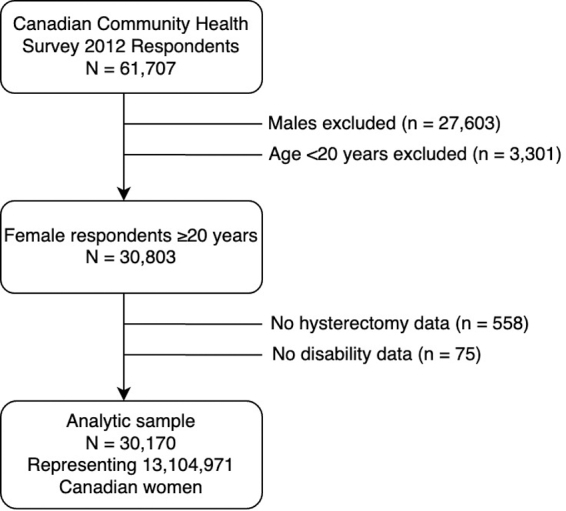
Selection of analytic sample from Canadian Community Health Survey 2012 respondents.

Our analytic sample consisted of 30,170 female respondents aged ≥20 years, representing 13,104,971 women in the Canadian household population (Fig. 1). Sample characteristics by (any) disability status are displayed in Table 1.

**Table 1.** Characteristics of Canadian Women Aged 20 and Older by Disability Status

	No disability Unweighted n* = 17,357 Weighted *n = 8,332,311		Any disability Unweighted n* = 12,813 Weighted *n = 4,772,660		
	%	95% CI	%	95% CI	Stand. diff
Age					
Childbearing, 20–44 years	52.1	50.7–53.6	29.6	28.0–31.3	47.1
Perimenopausal, 45–59 years	27.8	26.4–29.2	30.3	28.6–32.0	5.5
Postmenopausal, 60+ years	20.1	19.1–21.1	40.1	38.6–41.7	44.8
Income quintile					
5 (Highest)	20.8	19.7–21.9	13.9	12.8–15.2	18.2
4	19.9	18.8–21.0	16.2	15.0–17.6	9.5
3	20.4	19.3–21.6	19.5	18.3–20.9	2.2
2	19.0	17.9–20.2	23.6	22.2–25.0	11.1
1 (Lowest)	19.9	18.6–21.3	26.7	25.2–28.3	16.2
Education					
PS certificate	66.7	65.2–68.1	57.4	55.7–59.2	19.1
High school or some PS	23.4	22.2–24.8	23.7	22.2–25.3	0.6
Less than high school	9.9	9.1–10.7	18.9	17.6–20.2	25.8
Employment					
Full-time at a business	31.4	30.2–32.7	52.3	50.5–54.0	43.2
Part-time at a business	49.0	47.5–50.5	31.7	30.0–33.5	35.7
Self-employed	10.9	9.9–12.0	9.1	8.1–10.3	6.0
Not employed	8.6	7.8–9.6	6.9	6.0–7.8	6.7
Ethnicity					
White	75.0	73.5–76.5	81.9	80.2–83.5	16.9
Visible minority	25.0	23.5–26.5	18.1	16.5–19.8	
Marital status					
With partner	64.6	63.2–66.0	58.2	56.5–59.9	13.1
Divorced/widowed	13.8	12.9–14.8	24.8	23.4–26.3	28.2
Single, never married	21.6	20.4–22.8	17.0	15.7–18.3	11.8
Body mass index					
Underweight	3.8	54.7–57.7	3.4	2.7–4.2	2.3
Normal	56.2	54.7–0.6	41.2	39.5–42.9	30.4
Overweight	25.7	3.2–4.5	30.0	28.4–31.6	9.5
Obese	14.3	24.5–27.0	25.4	24.0–27.0	28.3
Mental health disorder					
No	92.3	13.2–15.3	75.4	73.9–77.0	46.9
Yes	7.7	91.5–92.9	24.6	23.0–26.1	

Estimates are weighted to represent the Canadian household population.

CI, confidence interval; PS, postsecondary.

Women with disability were generally aged ≥60 years (40.1%, 95% CI 38.6–41.7), whereas women without disability were predominantly aged 20–44 years (52.1%, 95% CI 50.7–53.6). Women with any disability were more likely to have a household income in the lower quintiles, have less than high school education, work full-time at a business. A slightly larger proportion of women with disability self-identified as White ethnicity. Second to being married or common law, women with disabilities were more often divorced or widowed, whereas women without disability were more often single or never married. Obesity and mental health disorders were more common in women with any disability.

Prevalence and severity of disability are displayed in Table 2. Overall, 36.4% of women reported any disability (95% CI 35.4–37.5), 28.7% reported a functional disability (95% CI 27.8–29.7), and 29.8% reported an activity-limiting disability (95% CI 28.8–30.8). Moderate disability was more common than severe disability across all disability types. Conditions underlying disability stratified by hysterectomy status for each age group are shown in Supplementary Table S2. Distribution of underlying conditions only differed in the perimenopausal age group; a greater proportion of women with hysterectomy reported disease/illness and injury as the source of their disability, whereas a smaller proportion reported aging and conditions existing since birth.

**Table 2.** Prevalence of Self-Reported Disability and Severity in Canadian Women Aged 20 and Older

	Prevalence (%)	95% CI
Any disability	36.4	35.4–37.5
Moderate	20.7	19.9–21.6
Severe	15.7	15.0–16.4
Functional disability	28.7	27.8–29.7
Moderate	17.0	16.2–17.9
Severe	11.7	11.1–12.3
Activity-limiting disability	29.8	28.8–30.8
Moderate	20.7	19.9–21.6
Severe	15.7	15.0–16.4

Estimates are weighted to represent the Canadian household population.

**FIG. 2.**
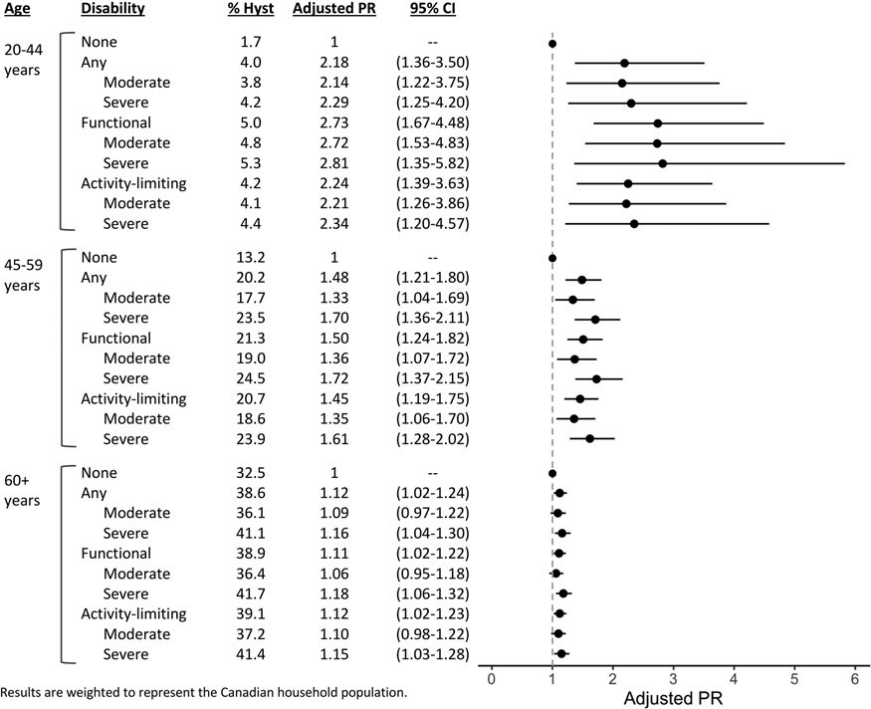
Association between disability and prevalence of hysterectomy stratified by age.

Prevalence of hysterectomy in the full sample was 15.4% (95% CI 14.7–16.1). Figure 2 shows the associations between self-reported disability and prevalence of hysterectomy stratified by age group and adjusted for income, education, ethnicity, marital status, and employment. Results from crude models are available in Supplementary Table S3; point estimates and statistical significance were similar between crude and adjusted models. Disability was significantly associated with higher prevalence of hysterectomy across all disability types and age groups. The associations were strongest in the youngest age group, and weakest in the oldest age group.

Among women of childbearing age (20–44 years), the prevalence of hysterectomy in women without disability was 1.7%, and the adjusted PRs were 2.18 (95% CI 1.36–3.50) for any disability, 2.73 (95% CI 1.67–4.48) for functional disability, and 2.24 (95% CI 1.39–3.63) for activity-limiting disability.

Among perimenopausal women (45–59 years), the prevalence of hysterectomy in women without disability was 13.2%, and the adjusted PRs were 1.48 (95% CI 1.21–1.80) for any disability, 1.50 (95% CI 1.24–1.82) for functional disability, and 1.45 (95% CI 1.19–1.75) for activity-limiting disability.

Among women of postmenopausal age (60 years and older), the prevalence of hysterectomy in women without disability was 32.5%, and the adjusted PRs were 1.12 (95% CI 1.02–1.24) for any disability, 1.11 (95% CI 1.02–1.22) for functional disability, and 1.12 (95% CI 1.02–1.23) for activity-limiting disability.

There was a slight gradient by disability severity, and this was consistent across all age groups and disability types. The adjusted PRs were larger for severe disability than for moderate disability; however, CIs were invariably overlapping. Results from our sensitivity analysis using multiple imputation with chained equations were similar to our main analysis (Supplementary Tables S4 and S5).

## Discussion

Using nationally representative cross-sectional data from Canadian women, we found that self-reported functional or activity-limiting disability was associated with significantly higher prevalence of hysterectomy. This association persisted after adjusting for demographic covariates and was consistent across disability types, severity levels, and age groups. The magnitude of this association was inversely related to age at data collection; the largest disparity was observed in childbearing-aged women, where the prevalence of hysterectomy was more than double in women with disability.

In the previous study on this topic that examined pre-existing disability and subsequent risk of hysterectomy, it was found that disparities in hysterectomy rates were concentrated in younger age groups and largely driven by the presence of multiple disabilities.^15^ Our finding of age-dependent trends and a strong association in the youngest age groups resemble those previously reported, although we were unable to address the role of multiple disabilities.

Age-dependent trends we report in this study should be considered in light of the lack of temporality with this cross-sectional data source. There is the possibility of reverse causality, whereby hysterectomy is associated with subsequent disability. Some,^23–25^ but not all,^26^ evidence suggests that women who have undergone hysterectomy (or surgical menopause) have a higher risk of functional limitations compared with women experiencing natural menopause or who are premenopausal. Authors of these previous studies acknowledged that observed effects may be heavily influenced by the extensive endocrine changes that occur in females during midlife, or may be explained by physical health conditions that preceded and gave rise to the decision for hysterectomy.^24,25^

More recent study has excluded women with functional limitations at study entry and observed a small (odds ratio ∼1.1) positive association between hysterectomy and new-onset limitations between ages 47 and 70 years that appeared to be largely attributable to concomitant bilateral oophorectomy.^27^ Taken together, there is limited evidence to suggest reverse causality is a major driver of our findings, particularly noting that our sample extends as young as 20 years. Hysterectomy is also associated with chronic conditions later in life, including increased risk of cardiovascular disease,^7^ osteoporosis,^28^ and certain types of cancer (*e.g.*, thyroid),^29^ many of which may be a source of disability.

Yet with this mechanism, one might expect our PRs to become larger as women age and conditions emerge, when in fact we noted the opposite. The more likely explanation for observed age-dependent trends is misclassification bias, where older women were more likely to experience onset of disability after (and possibly unrelated to) their hysterectomy thus biasing our estimates toward the null. Bearing this in mind, the strength of associations in the youngest childbearing-aged group is particularly concerning as indications for hysterectomy at this age would be largely menstrual in origin and have many pharmacological alternatives.^30–32^

Several lines of evidence support the interpretation that pre-existing disability may be linked to higher use of hysterectomy. Certain gynecologic conditions are documented to have higher prevalence in women with disability. Menstrual disorders are more common in women with epilepsy, diabetes, and autism,^33–35^ and endometriosis is frequently comorbid with autoimmune diseases.^34^ This risk elevation is modest at best, and unlikely to fully explain the observed associations. Hysterectomy for contraception or peripartum complications are more common in women with disabilities.^13,36^ Although these events are rare, they raise concern that women with disability may receive surgical intervention at lower threshold, possibly due to implicit provider bias.

Women with disability may experience unique obstacles managing gynecologic issues, which could impact treatment decisions. Physical and cognitive disabilities can make it difficult (or impossible) to independently maintain menstrual hygiene.^37,38^ Menstruation may also exacerbate symptoms related to disability, such as worsening of sensory and emotional issues related to disabilities such as autism.^35^ Women with arthritis have cited flare onset coinciding with certain points in their menstrual cycle.^39^ For a woman navigating disability and self-managing underlying health conditions, definitive amenorrhea may be an attractive option to eliminate the additional burden of a gynecologic issue, and they may perceive surgery as the best option to achieve this.

Health care experiences are also important to consider. Although most women report satisfaction with their decision to undergo hysterectomy, a considerable proportion report the scope of information from health providers regarding hysterectomy versus alternatives was suboptimal.^40^ This may be particularly problematic for women with disabilities who have unique needs during health care encounters. Appointment time spent maneuvering for the physical examination can detract from time remaining to discuss medical management, and communication or cognitive disabilities may impede their ability to fully engage in verbal discussions about treatment. Power imbalances may also play a role.

Studies on sexuality, contraception, and disability spotlight women's disempowerment and lack of confidence in advocating for their reproductive health.^11,41–43^ Women are cognizant of the complexity that their disability represents, and in some cases want to appear agreeable or compliant.^43^ Conversely, women may perceive that their health care provider lacks sensitivity to their needs or sufficient knowledge on treatment options in light of their underlying condition.^42^ These phenomena could result in women with disability being more likely to experience hysterectomy.

The foremost limitation of our analysis is the inability to establish temporality of disability preceding hysterectomy and lack of data on hysterectomy timing, indication (which generally vary by age),^44^ and concomitant oophorectomy; our detection of an association cannot be interpreted as evidence of causation. We used employment as a proxy for prescription drug coverage because outpatient pharmaceutical prescriptions are excluded from universal health care in Canada and instead often covered in employer-sponsored health benefits.

However, this proxy is likely imperfect, as quality of drug coverage is generally related to job sector with more skilled affluent positions providing superior benefits. Despite women with disability in our sample having high rates of full-time employment, they also tended to have lower education and income, which suggests they were less likely to hold skilled positions with high-quality drug coverage. CCHS respondents likely represent a range of physical disabilities but a subset of mild cognitive disabilities that permit living in a private dwelling and capacity to participate in a 45-minute survey.

Consequently, our findings are not generalizable to women with disability who require institutional living or have severely limited communication. The recency of our data source (collected in 2012) is a weakness, given the increasing popularity of hormonal intrauterine devices and introduction of new oral progestins, oral gonadotropin releasing hormone receptor agonists, and dermal contraceptive implants to the Canadian market in the past decade, which have broadened the number of hysterectomy alternatives. It is possible that the differences we observed may have narrowed in light of these advancements, or widened if access to newer pharmacological options is inequitable across disability status.

Our findings of a possible disparity in prevalence of hysterectomy by disability status evident in 2012 is thus foundational to document in support of the need for further investigation of this topic using contemporary data. More broadly, our study highlights the need for consistent and recent survey-based data on women's health in Canada, to document and track changes in health status and determinants over time.

Strengths of our approach include the large representative data source, and measurement of disability using self-perceived limitations as opposed to diagnostic coding from administrative data. The former aligns the WHO ICF by broadly capturing disability in the context of performing daily activities and participating in life situations, whereas the latter imposes a medical model in which disability is tied exclusively to having a diagnosis.^45^

## Conclusion

Prevalence of hysterectomy is disproportionately higher among women with disabilities compared with women without disabilities, with these differences most pronounced in women of childbearing age. Further research is needed to examine this trend outside of North America and to understand the chronology and drivers of these differences using prospective data and qualitative research. While awaiting future research on this topic, health care providers should be sensitive to recognizing disability in their patients, and ensuring they receive appropriate support to make informed decisions about medical management of gynecologic conditions.

## Supplementary Material

Supplemental data

Supplemental data

Supplemental data

Supplemental data

Supplemental data

## Abbreviations Used

CCHS: Canadian Community Health Survey
CI: confidence interval
ICF: International Classification of Functioning, Disability and Health
PR: prevalence ratio
PS: postsecondary
WHO: World Health Organization
