# Supplementary material for: Prevalence of Hysterectomy by Self-Reported Disability Among Canadian Women: Findings from a National Cross-Sectional Survey
Source: Womens Health Rep (New Rochelle). 2021 Nov 29;2(1):557–65. doi: 10.1089/whr.2021.0069 (PMC8665278; doi:10.1089/whr.2021.0069)
Supplement: Supplemental data [file Supp_TableS1.docx]

**Table S1.** Model equation and combination of coefficients for estimating age-stratified prevalence ratios from single model

| **Disability presence (binary)** | |
| --- | --- |
| Full Model | $\beta_{0}+\beta_{1}Disability+\beta_{2}Age1+\beta_{3}Age2+\beta_{4}Disability*Age1+\beta_{5}Disability*Age2+\beta_{6}Covariates$ |
| Childbearing (Age0) | $\frac{\text{p}_{\text{D1A0}}}{\text{p}_{\text{D0A0}}}=\beta_{1}$ |
| Peri-menopausal (Age1) | $\frac{\text{p}_{\text{D1A1}}}{\text{p}_{\text{D0A1}}}=\beta_{1}+\beta_{4}$ |
| Post-menopausal (Age2) | $\frac{\text{p}_{\text{D1A2}}}{\text{p}_{\text{D0A2}}}=\beta_{1}+\beta_{5}$ |
| **Disability severity (3-level categorical)** | |
| Full Model | $\beta_{0}+\beta_{1}Disability1+\beta_{2}Disability2+\beta_{3}Age1+\beta_{4}Age2+\beta_{5}Disability1*Age1+\beta_{6}Disability1*Age2+\beta_{7}Disability2*Age1+\beta_{8}Disability2*Age2+\beta_{9}Covariates$ |
| Moderate disability (Disability1) | |
| Childbearing (Age0) | $\frac{\text{p}_{\text{D1A0}}}{\text{p}_{\text{D0A0}}}=\beta_{1}$ |
| Peri-menopausal (Age1) | $\frac{\text{p}_{\text{D1A1}}}{\text{p}_{\text{D0A1}}}=\beta_{1}+\beta_{5}$ |
| Post-menopausal (Age2) | $\frac{\text{p}_{\text{D1A2}}}{\text{p}_{\text{D0A2}}}=\beta_{1}+\beta_{6}$ |
| Severe disability (Disability2) | |
| Childbearing (Age0) | $\frac{\text{p}_{\text{D2A0}}}{\text{p}_{\text{D0A0}}}=\beta_{2}$ |
| Peri-menopausal (Age1) | $\frac{\text{p}_{\text{D2A1}}}{\text{p}_{\text{D0A1}}}=\beta_{2}+\beta_{7}$ |
| Post-menopausal (Age2) | $\frac{\text{p}_{\text{D2A2}}}{\text{p}_{\text{D0A2}}}=\beta_{2}+\beta_{8}$ |
