# Supplementary material for: Prevalence of Hysterectomy by Self-Reported Disability Among Canadian Women: Findings from a National Cross-Sectional Survey
Source: Womens Health Rep (New Rochelle). 2021 Nov 29;2(1):557–65. doi: 10.1089/whr.2021.0069 (PMC8665278; doi:10.1089/whr.2021.0069)
Supplement: Supplemental data [file Supp_TableS2.docx]

**Table S2. Nature of condition underlying disability stratified by age group and hysterectomy status**

|  | **No Hysterectomy** | | **Hysterectomy** | | ***p-*value** |
| --- | --- | --- | --- | --- | --- |
|  | **%** | **95% CI** | **%** | **95% CI** |  |
| **Childbearing Age (20-44 years)** |  |  |  |  | **0.137** |
| **Injury** | **24.2** | **(21.3-27.3)** | **28.1** | **(15.4-45.7)** |  |
| **Disease/illness** | **28.6** | **(25.6-31.9)** | **37.8** | **(21.4-57.5)** |  |
| **Aging** | **3.8** | **(2.6-5.5)** | **9.1** | **(3.3-23.2)** |  |
| **Existed since birth** | **13.6** | **(11.7-15.9)** | **10.9** | **(4.8-22.7)** |  |
| **Other** | **29.7** | **(26.6-33.0)** | **14.1** | **(6.4-28.3)** |  |
| **Peri-menopausal (45-59 years)** |  |  |  |  | **0.041** |
| **Injury** | **19.4** | **(16.4-22.7)** | **23.2** | **(18.7-28.6)** |  |
| **Disease/illness** | **31.7** | **(27.7-36.0)** | **38.7** | **(33.2-44.5)** |  |
| **Aging** | **20.3** | **(16.7-24.5)** | **17.5** | **(12.7-23.6)** |  |
| **Existed since birth** | **8.7** | **(6.7-11.3)** | **5.1** | **(3.6-7.1)** |  |
| **Other** | **19.9** | **(16.6-23.6)** | **15.5** | **(12.3-19.5)** |  |
| **Post-menopausal (60+ years)** |  |  |  |  | **0.1683** |
| **Injury** | **11.1** | **(9.8-12.7)** | **12.2** | **(10.3-14.4)** |  |
| **Disease/illness** | **33.0** | **(30.5-35.7)** | **36.0** | **(33.1-39.0)** |  |
| **Aging** | **41.9** | **(39.0-44.8)** | **37.3** | **(34.5-40.1)** |  |
| **Existed since birth** | **6.1** | **(4.7-7.9)** | **5.6** | **(4.5-6.9)** |  |
| **Other** | **7.8** | **(6.6-9.2)** | **8.9** | **(7.4-10.7)** |  |
