# Supplementary material for: Prevalence of Hysterectomy by Self-Reported Disability Among Canadian Women: Findings from a National Cross-Sectional Survey
Source: Womens Health Rep (New Rochelle). 2021 Nov 29;2(1):557–65. doi: 10.1089/whr.2021.0069 (PMC8665278; doi:10.1089/whr.2021.0069)
Supplement: Supplemental data [file Supp_TableS3.docx]

**Table S3.** Crude association between disability and prevalence of hysterectomy across age groups

|  | Childbearing Age  (20-44 years) | | Peri-menopausal  (45-59 years) | | Post-menopausal  (60 years and older) | |
| --- | --- | --- | --- | --- | --- | --- |
|  | Crude PR | 95% CI | Crude PR | 95% CI | Crude PR | 95% CI |
| Any disability | 2.32 | (1.47-3.67) | 1.52 | (1.27-1.83) | 1.19 | (1.09-1.30) |
| Moderate | 2.24 | (1.30-3.87) | 1.34 | (1.06-1.68) | 1.11 | (1.00-1.24) |
| Severe | 2.49 | (1.39-4.47) | 1.78 | (1.45-2.18) | 1.26 | (1.14-1.40) |
| Functional disability | 2.86 | (1.77-4.63) | 1.57 | (1.31-1.87) | 1.18 | (1.08-1.29) |
| Moderate | 2.79 | (1.59-4.88) | 1.40 | (1.12-1.75) | 1.11 | (1.00-1.23) |
| Severe | 3.05 | (1.49-6.21) | 1.80 | (1.45-2.22) | 1.27 | (1.15-1.40) |
| Activity-limiting disability | 2.40 | (1.51-3.83) | 1.52 | (1.27-1.82) | 1.17 | (1.07-1.27) |
| Moderate | 2.35 | (1.36-4.04) | 1.36 | (1.09-1.70) | 1.11 | (1.00-1.23) |
| Severe | 2.53 | (1.32-4.83) | 1.76 | (1.42-2.17) | 1.24 | (1.11-1.37) |
