# Supplementary material for: Prevalence of Hysterectomy by Self-Reported Disability Among Canadian Women: Findings from a National Cross-Sectional Survey
Source: Womens Health Rep (New Rochelle). 2021 Nov 29;2(1):557–65. doi: 10.1089/whr.2021.0069 (PMC8665278; doi:10.1089/whr.2021.0069)
Supplement: Supplemental data [file Supp_TableS4.docx]

**Table S4.** Extent of missing covariate data

|  | N Observations | Weighted Proportion Missing | |
| --- | --- | --- | --- |
|  |  | % | 95% CI |
| *Covariates* |  |  |  |
| Age | 0 | – | – |
| Income | 515 | 0.3 | (0.3-0.4) |
| Education | 511 | 3.1 | (2.7-3.6) |
| Employment | 509 | 3.2 | (2.7-3.7) |
| Visible minority | 538 | 3.3 | (2.8-3.8) |
| Marital status | 53 | 0.3 | (0.2-0.4) |
| **BMI category** | **1141** | **5.9** | **(5.3-6.5)** |
| **Mental health disorder** | **62** | **0.1** | **(0.1-0.2)** |
| *Auxiliary variables* |  |  |  |
| Province | 0 | – | – |
| Physical comorbidities | 38 | 0.1 | (0.1-0.2) |
| Food security | 527 | 3.3 | (2.9-3.8) |
| Housing | 449 | 2.9 | (2.4-3.3) |
| Alcohol use | 250 | 1.5 | (1.2-1.9) |
| Smoking | 69 | 0.5 | (0.3-0.7) |
| Perceived health | <30 | – | – |

In accordance with Statistics Canada Guidelines for Tabulation, Analysis and Release,^29^ unweighted estimates are suppressed in instances where the cell size is less than 30 respondents. Auxiliary variables were province of residence, physical comorbidities (diabetes, high blood pressure, or heart disease), household food security (secure, insecure; derived by Statistics Canada using 18 questions on household food access over the past 12 months), smoking status (daily, occasionally, or none), alcohol use (regular, occasional, or none; derived by Statistics Canada using self-reported frequency of drinking in the past 12 months), perceived health (excellent, very good, good, fair, poor), and home ownership (yes, no).
