# Supplementary material for: Prevalence of Hysterectomy by Self-Reported Disability Among Canadian Women: Findings from a National Cross-Sectional Survey
Source: Womens Health Rep (New Rochelle). 2021 Nov 29;2(1):557–65. doi: 10.1089/whr.2021.0069 (PMC8665278; doi:10.1089/whr.2021.0069)
Supplement: Supplemental data [file Supp_TableS5.docx]

**Table S5.** Association between disability and prevalence of hysterectomy stratified by age group following multiple imputation with chained equations

|  | Childbearing Age  (20-44 years) | | Peri-menopausal  (45-59 years) | | Post-menopausal  (60+ years) | |
| --- | --- | --- | --- | --- | --- | --- |
|  | Adj PR | 95% CI | Adj PR | 95% CI | Adj PR | 95% CI |
| Any disability | 2.12 | (1.34-3.36) | 1.38 | (1.15-1.67) | 1.10 | (1.00-1.21) |
| Moderate | 2.09 | (1.21-3.62) | 1.26 | (1.00-1.58) | 1.06 | (0.95-1.17) |
| Severe | 2.21 | (1.24-3.96) | 1.56 | (1.27-1.93) | 1.15 | (1.04-1.28) |
| Functional disability | 2.65 | (1.63-4.28) | 1.42 | (1.18-1.70) | 1.09 | (1.00-1.19) |
| Moderate | 2.62 | (1.50-4.60) | 1.30 | (1.04-1.63) | 1.04 | (0.94-1.15) |
| Severe | 2.73 | (1.35-5.54) | 1.58 | (1.27-1.96) | 1.15 | (1.04-1.28) |
| Activity-limiting disability | 2.18 | (1.37-3.48) | 1.38 | (1.15-1.65) | 1.09 | (1.00-1.19) |
| Moderate | 2.16 | (1.25-3.73) | 1.27 | (1.02-1.60) | 1.06 | (0.96-1.18) |
| Severe | 2.24 | (1.18-4.28) | 1.54 | (1.24-1.91) | 1.13 | (1.02-1.25) |
